# Supplementary material for: Respectful family planning service provision in Sidama zone, Southern Ethiopia
Source: PLoS One. 2020 Sep 4;15(9):e0238653. doi: 10.1371/journal.pone.0238653 (PMC7473780; doi:10.1371/journal.pone.0238653)
Supplement: S1 File — (DOCX) [file pone.0238653.s001.docx]

**Family planning service provider related questionnaire Adopted from RMC training guideline implemented by Ethiopian Midwives Association.**

| **Socio-demographic questioner** | |
| --- | --- |
| **Question** | **Possible option/s** |
| Name of health institution |  |
| Age in years? | ________________________ |
| Sex | 1. Male B. Female |
| Marital status? | A. Single  B. Married D. Widowed E. Divorced |
| What is your religion? | A. Orthodox  B. Protestant  C. Muslim  F. Other (specify)_______________________ |
| Average monthly income? |  |
| Qualification | A. Diploma B. First degree C. Masters  D. Others (Specify) |
| Profession | 1. Health Assistant 2. Nurse 3. Midwifery 4. Health Officer 5. Medical Doctor 6. Others– Specify |
| Year of Experience |  |
| Ever trained on respectful maternity care? | 1. Yes B. No |
| Satisfied with current work environment | 1. Yes B. No |

**Knowledge questions**

**Instructions:** Write the letter of the single **best** answer to each question in the corresponding blank.

1. Respectful Maternity Care is: ______

- 1. Is a global problem
  2. Occurs in low, medium and high income countries
  3. Is a violation of human rights
  4. a and b
  5. All of the above

2. Some examples of respectful maternity care include: ______

1. Speaking to the woman in her own language
2. Allowing woman to leave the facility even if she has not paid her bill
3. Protecting the woman from information about herself, her condition and her care
4. a and b
5. All of the above

3. Choice of companion during family planning: ______

1. May be a good idea, but has never been shown scientifically to improve maternal or neonatal outcomes
2. Is advised in birthing centers but concerns about hygiene mean it is not appropriate in busy hospitals
3. Is an example of respectful maternity care
4. a and c

4. Respectful maternity care means that: _______

- 1. Women have access to hospitals and doctors for primary care
  2. Women are protected from information about themselves or their care when danger signs, or dangerous conditions, appear
  3. Women are empowered to become active participants in their care
  4. a and b E. a and c F. All of the above

5. Which of the following facilitate health care workers to provide respectful maternity care?

- 1. supportive supervision and management
  2. Information, training, and development
  3. adequate supplies, equipment, and infrastructure
  4. all of the above

6. Characteristics of Healthcare provider to be avoided is/are?

- 1. Centered on the professional and not on the woman and her family
  2. Impersonal
  3. Family unit separated during labor and birth
  4. Empowerment of the woman
  5. b,c and d f. a, b and c

7. Which one of the following is/are not Potential Contributors to Disrespect and Abuse in Facility-Based Childbirth?

- 1. Financial barriers
  2. lack of women‘s autonomy and empowerment
  3. Normalization of disrespect and abuse during childbirth
  4. Evidence-based clinical care standards
  5. all of the above

8. Which one of the following is not approach to respectful maternity care?

- 1. Use the universal rights of childbearing women
  2. Incorporate respectful maternity care as a standard of practice
  3. Support healthcare providers to deliver non-Dignified Care
  4. all of the above

9. Performance standard help to _____

- 1. Provide the health care provider with specific performance expectations for each major duty
  2. Improve the performance and quality of health services
  3. Promoting a culture of continual measurement of performance based on objective standards of care
  4. Assess and measure the individual performance.
  5. all of the above

10. Rough treatment, shouting, and publicly divulging private patient information are example of ___________care?

1. Discrimination
2. Physical abuse
3. Non-Dignified care
4. Non-consented care

11. Which of the following is not providers prejudice in giving quality of care?

1. Discriminatory behavior against certain sub group based on HIV/AIDS status
2. Mistreatment based on ethnicity
3. Good treatment linked to low economic status
