# Supplementary material for: Respectful family planning service provision in Sidama zone, Southern Ethiopia
Source: PLoS One. 2020 Sep 4;15(9):e0238653. doi: 10.1371/journal.pone.0238653 (PMC7473780; doi:10.1371/journal.pone.0238653)
Supplement: Attachment — Submitted filename: Response to reviwers.docx [file pone.0238653.s002.docx]

**To:**	*The Editor*

*PLoS ONE.*

**Re:**	*Submission of Modified manuscript*

**Manuscript title:** *Respectful family planning service provision in Sidama zone, Southern Ethiopia***.**

**Authors:** Melese Siyoum, Ayalew Astatkie, Zelalem Tenaw, Abebaw Abeje, Teshome Melese

Dear editor,

Thank you a lot for the constructive comments furnished by the peer reviewers of your esteemed journal. We have given due consideration to all the comments and suggestions and have revised our manuscript accordingly. We have addressed the specific issues raised and our point-by-point response to the comments is given below.

Sincerely,

The authors

**Manuscript title**: Respectful family planning service provision in Sidama zone, Southern Ethiopia

| **Comments from Reviewer #2** | **Response** |
| --- | --- |
| **Abstract section:**   1. Introduction part lacks information about family planning 2. Result section is written awkwardly 3. Conclusion session lacks language consistence | Thank you a lot for raising these issues.  All comments accepted and corrected.  The lack of information on family planning in the introduction is because there is lack of literature regarding respectful care among family planning clients. Now, we have described about poor client-provider interaction at family planning units based on available literature.  The whole document has now been thoroughly revised for language. The conclusion sections is also corrected as per the comment. |
| 4. Background session missed important references | Thank you for this comment. We have done the required revisions.  The lack of information on family planning in the background section was because of the lack of literature related to respectful care among family planning service users. Now, we have incorporated evidence from the available literature about family planning in the introduction (see reference 25).  A description of the gap of measuring disrespectful care at family planning unit using tool developed for maternity care is also provided. |
| 5. Unique Importance of the study need to be strengthened | **Thank you again**  Importance of the study for family planning programme is well described at the end of background session. |
| 6. Rationale for the selection of the Mothers on Respect index (MORi) could be explained in greater detail | Thank you. Now we have described it in more detail.  This tool was preferred than the others because the previous tools were confirmed to have a gap in assessing respectful care at FP ( see ref 25).  In addition, the previously used tool (developed by Bohren et al., was not validated). The current tool we used is validated in USA and Canada.  It can be easily used in family planning context since the focus area was on autonomy (decision making), culture and client-provider interaction. |
| 7. Questionnaire for service providers were not well described | Thank you for pointing this out. Now we have provided a clear description as per the comment.  The questionnaire used to assess service providers’ related factors were adapted from the training manual on respectful maternity care (see ref.23) developed by the Ministry of Health of Ethiopia and Ethiopian Midwives Association. |
| 8. Table2: some of the variables were not clear and need rephrasing | **Thank you. Now it is Corrected** as:   a. Service provider =Family planning service providers b. Involved in decision making = involved client in decision making |
| 9. Interpretation of the finding needs more description. What is “highly respected mean?” | Thank you for pointing this out. Now we have thoroughly revised the description and interpretation of the results in the results and discussion sections. We hope now the interpretations would make more sense.  Further, one advantage of the MORi tool is to show the extent to which the client is respected. Previously respectful care was measured as either 100% or 0% based on whether the client missed one component of care or not. This study shows to what extent the client received respectful care.. |
| 10. Level of significance 0.2 seems too high. | Thank you for raising the issue.  In this study Significance level <0.2 was used for binary analysis, it is not for the final analysis (multivariate). |
| 11. Discussion section doesn’t describe implication of the finding | Thank you. Revised accordingly.  The implication of the finding is included at the end of discussion and conclusion sections. |
| 12. Discussion section needs major revision for English and write up | Thank you for pointing this out. Now we have thoroughly revised the discussion section.  The overall write up and language was edited by a pre-publication support service (PREPSS) from Michigan University. |
| 13. Limitation section and conclusion part need to be more elaborated | Thank you for this comment. Done accordingly.  Elaborated by including the implication of the findings. |

| **Comments from reviewer #1** | **Response** |
| --- | --- |
| **Background**   1. Rationale of this study need to strengthen by honing on family planning related problems. | Thank you for raising this very important issue. Now we tried to strengthen it by incorporating important points**.**  Poor client-provider interaction at family planning unit was described. Additional references were added. The gap of measuring disrespectful care at family planning unit using tool developed for maternity care was clearly stated. Moreover, lack of evidence on family planning is incorporated (see ref 25).  Importance of the study for family planning programme is well described at the end of back ground session. |
| 2. Clarify whether the binary or six scale questionnaire was adopted from MORi tool? | Thank you again.  It was **described** under methodology section that we used the six scale tool because it was confirmed that the six scale tool well measures the level of respectful service provided. |
| 3. Describe the changes(modifications) made to the questionnaire | Thank you very much.  The only modification made to the tool was that we modified “maternity care” in to “family planning service”. It can be easily used in family planning context since the focus area was on autonomy (decision making), culture and client-provider interaction. |
| 4. The base for operational definition is not clear | Thank you again.  We attached here a one page of the MORi tool as supporting file. It clearly state how to classify the level of respectful care (see S1). |
| 5. Sample size determination is not clear as whether the proportions were taken from two populations. | Thank you for raising this issue,  Yes the proportions were taken from two populations. Respectful service provision was 87% and 80% by trained and untrained providers in Kenya. Now we have corrected the description as per the comment. |
| 6. Why the proportions were taken from study in Kenya while proportion were reported from Ethiopia. | Thank you for this question.  This study was needed for intervention to improve respectful family planning service by Center of International Reproductive Health Training (CIRHT) Ethiopian branch. So we preferred to use proportions from interventional studies. Currently different interventions are being implemented in this study area and its impact will be assessed near the future. |
| 7. It is not clear how 40 health facilities were selected | Thank you for this comment. Now we have clearly stated in the methodology section how the 40 health facilities were selected.  These 40 health facilities were selected randomly from all health facilities in the zone. |
| 8. Number of service providers participated | Now it is clearly stated  All 66 family planning service providers during data collection period were included. |
| 9. Table1: cut off point for under-poverty is needed | Thank you for raising this important question**.**  Now we described it within the table and under operational definition sections.  Income was categorized as under-poverty if their average daily income is equivalent to <1.25$. |
| 10. Table2: service providers were not specifically described | Thank you. Now it is corrected  Service provider means Family planning service providers by the time of data collection. |
| 11. Table3: clarify “satisfied provider”. | Thank you. Now it is clearly described.  It is service provider’s perceived satisfaction with their current status. |
| 12. Interpretation for relationship between respectful care with short acting methods as well as with provider gender needs caution with such skewed distribution of the raw data | Thank you very much for raising this important issue.  As you see it from the regression table, the finding is significant with acceptable confidence interval. However, the skewed distribution of the raw data is now described as a limitation. |
| 13. The manuscript need edition for language clarity. | Thank you for pointing this out. Now we have thoroughly revised the discussion section.  The overall write up and language was edited by a pre-publication support service (PREPSS) from Michigan University. |
